# “Maskandi experience”: exploring the use of a cultural song for community engagement in preparation for a pilot Sterile Insect Technique release programme for malaria vector control in KwaZulu-Natal Province, South Africa 2019

**DOI:** 10.1186/s12936-021-03736-9

**Published:** 2021-04-28

**Authors:** Pinky N. Manana, Sara Jewett, Jabulani Zikhali, Dumsani Dlamini, Nondumiso Mabaso, Zothile Mlambo, Roxanne Ngobese, Givemore Munhenga

**Affiliations:** 1 Centre for Emerging Zoonotic and Parasitic Diseases, National Institute for Communicable Diseases (NICD), Division of National Health Laboratory Services (NHLS), Johannesburg, South Africa; 2 Wits Research Institute for Malaria, School of Pathology, Faculty of Health Sciences, University of the Witwatersrand, Johannesburg, South Africa; 3 School of Public Health, Faculty of Health Sciences, University of the Witwatersrand, Johannesburg, South Africa; 4 Clinton Health Access Initiative, Malaria, KwaZulu-Natal, South Africa; 5 Department of Health, Environmental Health, Malaria and Communicable Disease Control, KwaZulu-Natal, South Africa

**Keywords:** Sterile insect technique, Malaria control, Community engagement, Cultural song, KwaZulu–Natal Province, South Africa

## Abstract

**Background:**

An assessment of the Sterile Insect Technique (SIT) as a complementary malaria vector control tool, is at an advanced stage in South Africa. The technique involves the release of laboratory-reared sterilized male mosquitoes of the major malaria vector *Anopheles arabiensis*, raising social, ethical and regulatory concerns. Therefore, its implementation largely depends on community participation and acceptance. Against this background, it is critical that robust and effective community strategies are developed. This study describes the development of a cultural song to engage the community and increase awareness on SIT and malaria control in KwaZulu-Natal, South Africa.

**Methods:**

An exploratory concurrent mixed-methods study was conducted to get opinions about the effectiveness of a cultural song developed to engage communities and increase acceptability of the SIT technology. Two self-administered surveys (expert and community) were conducted. Additionally, more in depth opinions of the song and its effectiveness in conveying the intended information were investigated through three community dialogue sessions with community members in the study area.

**Results:**

A total of 40 experts and 54 community members participated in the survey. Four themes were identified in relation to the appropriateness and effectiveness of the song, with a fifth theme focused on recommendations for adaptations. Overall, the song was well received with the audience finding it entertaining and informative. Responses to unstructured questions posed after the song showed an increase in the knowledge on malaria transmission and SIT technology. In particular, the explanation that male mosquitoes do not bite allayed anxiety and fears about the SIT technology.

**Conclusion:**

The song was deemed both culturally appropriate and informative in engaging community members about the SIT technology. It proved useful in promoting health messages and conveying SIT technology as a complementary malaria vector control tool. With minor adaptations, the song has potential as an area-wide community engagement tool in areas targeted for sterile male releases.

**Supplementary Information:**

The online version contains supplementary material available at 10.1186/s12936-021-03736-9.

## Background

Malaria is a public health challenge worldwide, with approximately 228 million cases and 405 000 deaths reported in 2018 [1]. More than 90% of malaria cases and deaths were reported from Africa, with sub-Saharan Africa being the most affected region [2]. In 2018, the incidence of malaria was estimated at 57 cases per 1,000 population [1]. The disease impacts negatively on the social, health and economic lives of affected people [3]. Although significant progress has been made in reducing the malaria burden in South Africa (SA), sporadic outbreaks still occur in the low-altitude northern and north-eastern regions of the country’s three endemic provinces (Limpopo, Mpumalanga, KwaZulu-Natal). In these endemic regions, about 4.3 million people are at risk [3, 4].

Success in reduction of malaria transmission in SA is largely attributed to organised vector control achieved through Indoor residual spraying (IRS) of households with dichloro-diphenyl-trichloroethane (DDT) and pyrethroids. This approach has been in operation for over seven decades [5, 6]. However, although IRS remains effective, it faces a number of challenges including insecticide resistance in targeted vector populations, outdoor biting populations that are not amenable to IRS, environmental concerns over continual use of insecticides and the high economic cost of using insecticides in low malaria transmission settings [7, 8]. Against this background, additional vector control interventions are needed to supplement the current strategy if the South African government is to meet its mandate to eliminate malaria by 2023 [9]. One such strategy under investigation is the Sterile Insect Technique (SIT) [10].

Feasibility studies on the applicability of SIT against the major malaria vector *Anopheles arabiensis* are ongoing [11]. Significant progress has been made in various aspects of the SIT packages including mass rearing, sterilization/mating compatibility and competitiveness, and development of sex separation systems [6, 11, 12]. This has paved the way for open field trials. To implement field trials successfully it is critical that cohesion between the community and the project is established. According to the World Health Organization (WHO)[13], community engagement is central to any public health intervention. This is particularly so for the SIT technology that hinges on the release of laboratory-altered mosquitoes. The release of mosquitoes into communities raises social, ethical and regulatory concerns [14]. Therefore, there is a need to characterize and robustly address such concerns before field trials can be initiated. It is important to engage and increase awareness about the programme in the target communities to ensure adequate cooperation and participation. Previous studies have demonstrated that directly engaging the community plays an important role in improving the acceptability and effectiveness of programmes [10, 15–18].

Community engagement is defined as working with all relevant partners who share the same interests to foster meaningful research; and collaboration to achieve common goals, including being sensitive to community contributions so as to protect their beliefs [19]. It involves supporting those affected to understand the risks they face, and empower them in making informed actions [13]. Community engagement efforts should be mindful of differences in culture, ethics, customs, and social structure between populations [17, 20]. Any programmes that fail to consider beliefs and perceptions of the community face negative attitudes or practices [17], cultural impositions [21], introjection [22], and symbolic violence ultimately leading to failure in achieving the intended goals [10].

Several strategies for communication and engaging communities are available. Different countries choose to use different methods based on the most effective strategies applicable under their local context. In sub-Saharan Africa, use of artistic forms [23–26] to engage with communities is the most commonly reported strategy. According to Bunn and colleagues [24], 17 different categories of art forms are widely used in sub-Saharan Africa with theatre, music and song, TV/radio, visual arts and storytelling being among the top 5.

Music and song are used broadly, especially during advertising. Music significantly increases the ability of audiences to understand and remember a concept [27]. This is often done through “jingles” and repetitions [27]. Previous studies [28, 29] have reported the use of music to promote behavioural change in adolescents and adults to help with disease prevention and management. Music, song and dance were used in Africa for HIV/ AIDS and Ebola promotional messages, to improve preventative interventions for these diseases [25, 30–37]. During these community engagement campaigns, researchers either worked with popular artists or musicians to compose songs that were used or worked with communities to develop the songs. Specific to malaria, a song in Gambia was used successfully to encourage bed net repair for malaria prevention [38]. A study done in SA [39, 40] in Zulu-speaking communities reported that music provided support networks for people and a platform for preventive communication.

“Maskandi” is a type of traditional Zulu music that is popular and mostly played in KwaZulu-Natal (KZN) Province because of its richness in the Zulu heritage and importance to the Zulu tribe [41]. It has been in existence for many years and evolved within South African society. In an evaluation of how this indigenous music genre could be used as a tool for improving literacy, Ntombela [41] revealed a number of socio-cultural themes embedded in “Maskandi” music. These include use of metaphoric expressions, call-and-response patterns, repetition, indirection, interactive and group dynamics. These are general, yet particular, to the isiZulu tradition [41]. Against this background, “Maskandi” is a potential medium that can be used in KZN to engage the community for any new public health intervention. This study assessed the acceptability of using a cultural “Maskandi” genre song to engage with communities, increase awareness and knowledge on malaria control with specific focus on an area targeted for pilot of sterile male mosquito releases in Jozini, KZN, SA.

## Methods

### Study design

**Fig. 1:**
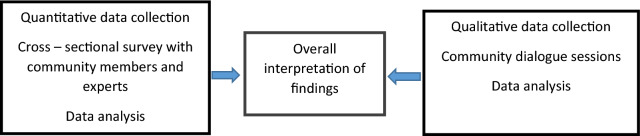
Triangulation/ exploratory concurrent mixed methods study design employed to assess acceptability, feasibility and appropriateness of the song in Jozini, KwaZulu-Natal Province, South Africa

The study used an exploratory concurrent mixed method design that included both quantitative and qualitative approaches (Fig. 1).

### Study setting

**Fig. 2:**
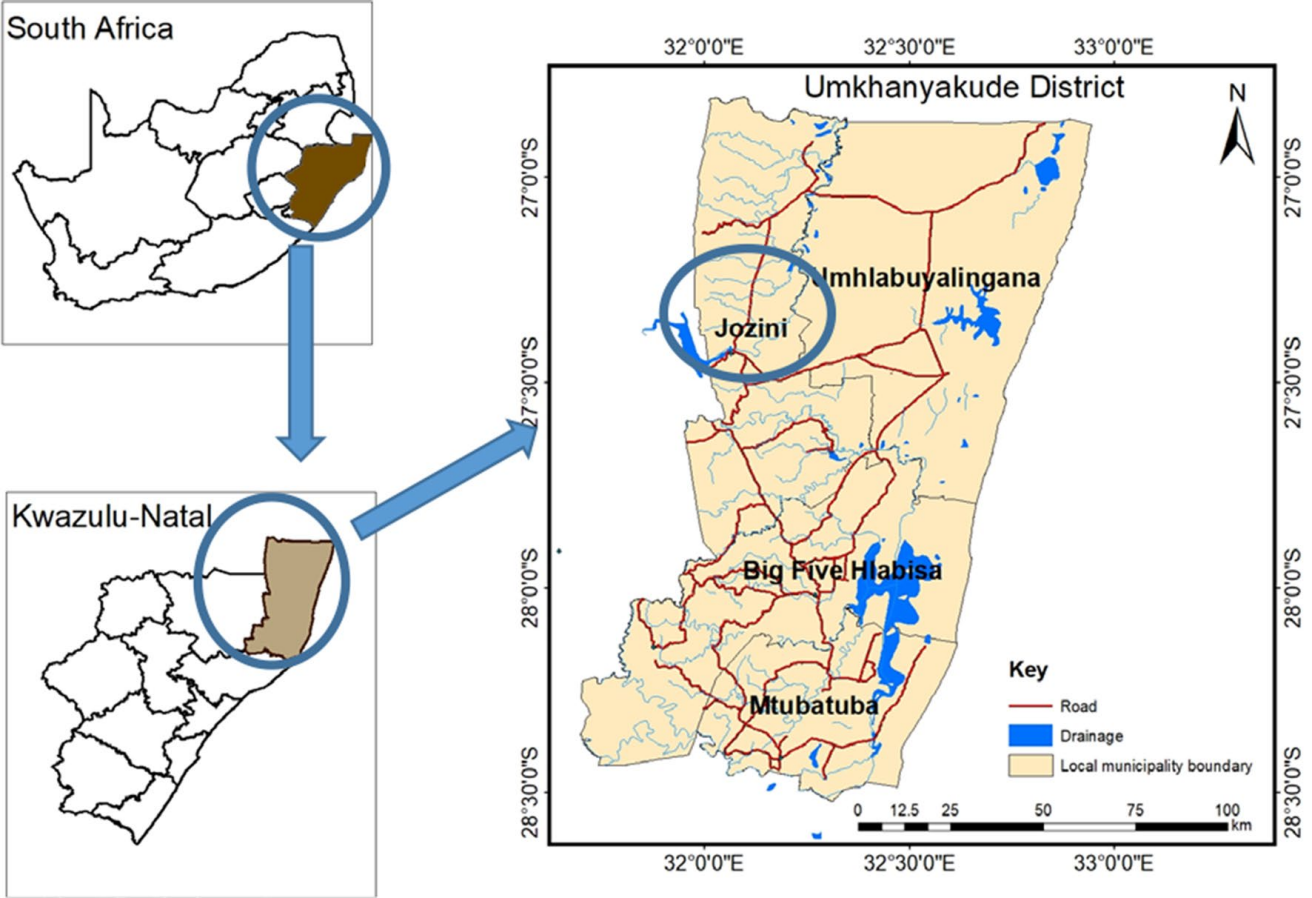
Map of South Africa showing the study area, Mamfene in Jozini local municipality, KwaZulu-Natal Province, South Africa

The study was conducted in Mamfene, Jozini local municipality, KZN. Jozini local municipality is part of uMkhanyakude District Municipality and is located in northern KZN, sharing boarders with Swaziland and Mozambique (Fig. 2). The Jozini local municipality is estimated to be 3 442m^2^. It is the most populated municipality within the uMkhanyakude district municipality with an estimated population of 198 215 [42]. The total number of households is 44 584. Jozini’s population is young, with 65% of the people aged below 25. The 0–14 year olds constitute 41.5% of the population and 15–24 years constitute 23% of the population. Adults, aged 65 and above constitute only 3.4% of the total population. Females constitute 54% of the population.

The Mamfene area comprises 10 sections, three of which serve as SIT sentinel entomological surveillance sites. The population in the three sections is: “Methods Section” (n = 2024); Sect. 8 (n = 4592) and “Data collection Section” (n = 2167) [42]. The community of Mamfene uses three health care facilities. There is also one community hall, two churches, twenty-one secondary schools, five primary schools, four colleges and one community radio station.

### Song development

A “Maskandi” group known in the community was approached to assist with composing a song on SIT technology. The group was provided with a pamphlet summarizing information on malaria and SIT technology as a guide to develop lyrics and music for the song in IsiZulu (local language). The translated lyrics can be found in Additional file 1. The song was first played during the World Malaria Day event commemorated in the study area in April 2019. The song was concurrently audio-recorded and used for data collection in this study.

### Study populations and sampling

The primary population of interest for the study was the Mamfene community, which is the target area for a pilot sterile male release programme. The campaign targeted two age groups; 18–49 year olds (considered as younger age group) and 50 years and above (considered as older age group). This was informed from a Malaria Knowledge, Attitudes and Practice (KAP) survey conducted in 2015 in Jozini [43]. The inclusion criteria were adults over 18 years old and residing in either Mamfene “Methods Section”, “Questionnaire administration Section” or “Data collection Section”, who were willing to participate and had given their consent to participate. To gather data, participants were invited to listen to a song followed by completion of a self-administered questionnaire. Furthermore, adults with the same inclusion criteria were also invited to community dialogue sessions.

The secondary study population was experts with experience in malaria control, who had an understanding of the local language and culture, including teachers in the study area. This included the malaria control programme teams, Environmental Health Practitioners, Information Education and Communication (IEC) teams, and Indunas (headman). Lastly, medical scientists were recruited as experts based on their knowledge of science and understanding of the SIT technology.

### Questionnaire development

The community questionnaire was divided into two sections: demographics and community engagement questions. The questionnaire was adapted from a tool used by Anderson et al*.* [44]. The questionnaire was translated into the study area local language (IsiZulu) by an expert who is fluent in both English and IsiZulu, and piloted with an IsiZulu speaking colleague at the National Institute for Communicable Diseases (NICD), originally from KZN. The first author, also fluent in IsiZulu and English, verified the translated version with the original questionnaire to ensure that the main idea was not misunderstood. The piloted questionnaire was administered at different time points for participants in the different communities. The community survey had mixed (closed and open-ended) questions (Additional file 2) because there was a need for more information from the community regarding their perception, knowledge and understanding of the song.

The expert survey was structured similarly to the community survey, but was self-administered in English. The experts’ survey used a 5-point Likert—scale to rank their opinions on the lyrics and the song (Additional file 3).

### Questionnaire administration

Young participants completed the self-administered part of the survey, while interviewers supported some older participants who found this challenging. The second part of the questionnaire was interview-administered for everyone. Before questionnaire administration; project aims and study procedures were clearly explained and discussed with the participants. For both the community and expert surveys, an audio recording of the song was played once, for approximately 10 min, after which study participants completed the questionnaires. English subtitles were included for the experts as some of them were not conversant in IsiZulu, which were checked by the first author who is fluent in both languages.

### Data collection

Data collection was done twice, first a community survey in November 2019 that was conducted immediately after a routine monthly community meeting with 100 community members attended and, secondly, an experts’ survey done in January 2020.

For the community survey, a total of 54 purposefully selected participants from the study area completed the self–administered questionnaires, with interviewers supporting any participants who were struggling with completing the self–administered questionnaires. Immediately after completing the surveys, all community members were invited to stay for community dialogue sessions (CDS) [45, 46]. The CDS were facilitated by the first author and trained field workers, using the questionnaire themes as a guide. These sessions were audio–recorded and field notes were taken. The CDS explored acceptability, cultural appropriateness of the song and the feasibility of using a song as an engagement strategy.

For the experts, information was collected through the self–administered questionnaire with 40 experts’ participating in the survey.

### Data management and analysis

Quantitative data were captured in RedCap and exported to Excel. Open response answers from the community survey were also exported from RedCap to an Excel file for post-hoc coding. Descriptive statistical analysis was conducted using STATA Version 15. Measures of central tendency and dispersion were calculated for quantitative variables and proportions were calculated for categorical variables. Frequency distribution tables were developed to show differences in the relative frequencies of variables.

Audio recordings of the CDS with the community members were downloaded from the tape recorder and stored on a secure office computer. Data were firstly transcribed verbatim and then translated from IsiZulu to English. The transcripts were saved in a Word file, where they were coded manually. A hybrid coding approach was conducted by the first two authors to establish themes as described by Fereday et al*.* [47]. The steps include familiarisation of data by listening to individual audio recordings and reviewing of transcripts and field notes; reviewing and coding of sections of data transcripts using both inductive and deductive approaches matched to particular themes in the questionnaires, developing a working analytic framework, charting and summarising coded data in a table, analysis and interpretation of themes. Selected participants’ quotes from each theme generated during analysis are presented.

As a final step, triangulation of both quantitative data and qualitative themes was conducted to identify any inconsistencies and to present a more integrated picture of the song’s appropriateness. By drawing on multiple sources, triangulation enabled the validation of results. Where data did not agree, authors reported on all perspectives and then discussed the implications based on the broader literature.

## Results

### Characteristics of study participants

A total of 140 individuals participated in the study, 100 community members and 40 experts. Of the 100, 54 participated in the community survey and all (100) participated in the community dialogue sessions. The experts’ survey had 40 participants. Their characteristics are summarized in Table 1.

**Table 1:** Demographic characteristics of study participants during the assessment of acceptability of a cultural song as a community engagement medium in KwaZulu-Natal Province, South Africa

Characteristics of study participants	Expert survey N = 40 (%)	Community survey N = 54 (%)	Community dialogues N = 100 (%)
Age (in years)			
Median age	36 (23–66)	43 (18–78)	43 (18–78)
Gender			
Female	29 (72)	37 (69)	69 (69)
Position/Role			
Scientist	26 (65)	N/A	N/A
Malaria control programme	11 (27)	N/A	N/A
Educator	3 (8)	N/A	N/A
Induna (headsman)	N/A	N/A	2 (2)
Heads of households	N/A	17 (31)	40 (40)
Unemployed	N/A	36 (67)	47 (47)
Some form of education	N/A	35 (65)	60 (60)

The recruited (n = 54) community members completed the community survey and responded to all questions in the questionnaire. The majority of these participants were females (69%; n = 37). Ages of the participants ranged between 18 and 78 years old, with a median age of 43 years. Heads of household accounted for 31% (n = 17) of the participants. The majority of the participants (68%, n = 36) were unemployed and 65% (n = 35) of the participants had some form of basic education. A total of 76% (n = 41) participants were from “Data collection Section Ebiva), followed by “Questionnaire administration Section” with 15% (n = 8) and “Methods Section with 9% (n = 5).

All 100 community members participated in the community dialogue sessions. The age range of participants was between 18 and 78 years with 69% being females. Participants included mothers, fathers, grandparents, youth and caregivers. A total of 40 experts completed the self-administered questionnaire. Of these, 65% (n = 26) were medical scientists, 27% (n = 11) were malaria control programme members and 8% (n = 3) were educators. The majority of the participants were female 72% (n = 29). The median age was 36, with an interquartile range of 23—66.

Four cross-cutting themes relating to the song’s appropriateness were identified and explored during both the surveys and CDS. These included the song’s cultural appropriateness, content appropriateness, appropriateness of delivery and overall appropriateness. Table 2 summarizes individual questions relating to each of these themes and open-ended responses as well as CDS.

**Table 2:** Expert and community responses to survey questions categorised by themes during the assessment of acceptability of a cultural song as a community engagement medium in KwaZulu-Natal Province, South Africa

Themes	Experts survey n = 40 (%)	Community survey n = 54 (%)
Cultural appropriateness		
1. Are the words in the song culturally appropriate for Jozini?	30 (75)	54 (100)
2. Are the words in the song age appropriate for Jozini?	32 (80)	53 (98)
Delivery appropriateness		
3. Are the words in the song clear and is it easy to understand what is being said?	34 (85)	52 (96)
4. Is the song easy to remember and does it allow people to engage?	34 (85)	53 (98)
5. Is repetition of verses in the song useful and can people benefit from it?	34 (85)	54 (100)
Content appropriateness		
6. Is information on malaria in the song useful and adequate?	37 (92)	50 (92)
7. Is informing people that mosquitoes are the vector for malaria an appropriate way to engage?	39 (97)	*
8. Is informing people that male mosquitoes do not bite a useful way to engage?	34 (85)	*
9. Is informing people that SIT is an additional vector control method appropriate and useful	35 (87)	*
10. Is informing people that SIT will help target outdoors mosquitoes appropriate and useful?	32 (80)	*
11. Is the information in the song enough to help people understand SIT?	32 (80)	52 (96)
Overall appropriateness		
12. Is the song appropriate to engage with the community on SIT?	36 (90)	54 (100)

^*^Question not asked in a closed-ended format in community survey

### Cultural appropriateness of song

All community members (100%) agreed that the song was culturally appropriate for Jozini community, as did 75% of the experts. In the CDS, many community members highlighted that the genre with ‘an African beat’ and use of isiZulu as elements made the song appropriate for intended use.

*“We love this genre of music called* “Maskandi” *a lot and the song warned us about malaria.” (Group 1, Female, 60).*

“The song is perfect and having it in IsiZulu is really helping in making us understand malaria. It is also good that the singer is a local resident.” (Group 2, Female, 49)

On age appropriateness of lyrics in the song, all community members answered in the affirmative while 80% of experts agreed on the suitability of the lyrics for the targeted age groups. However, there were mixed views during CDS. Some opinions aligned with the survey:

“This song is appropriate for all age groups and it warns us about the signs and symptoms of malaria.” (Group 2, Female, 49)

However, other community members thought the song genre was more attractive to older listeners. One 26-year old female community member from Group 1 explained that it was less the content than the genre she found inappropriate:

“The song is very informative and warns us about malaria. However, I do not enjoy this type of music.”

This opinion was however not shared by all young people, with a 28 year old female from Group 3 exclaiming: *“This song has great rhythm and I can dance to it.”*

### Appropriateness of song delivery

A variety of questions were asked regarding the song format. Over 95% of community members reported that the words were clear and easy to understand, that the message was easy to remember and that the use of repetition supported engagement. While community feedback was mainly positive, a few people highlighted issues around the aesthetic delivery of the song. For instance, one participant complained:

“The song is not clear enough. I did not get everything that the song is about, especially the backing vocalists.” (Group 1, Female, 32)

Most negative comments about the song delivery focused on audibility, particularly towards the end of the song. While the majority of experts gave positive responses regarding the delivery of the song, 85% of experts responded in the affirmative to all the three questions on song delivery, while there were more who expressed concern on the delivery of the song when compared to community members. One expert concluded, *“The song is good, easy to understand, affirmative and appropriate.”* (Scientist and MCP staff) However, another expert expressed frustration: *“The song is too long, too much repetition.”* (MCP staff)

This concern was however not echoed by any of the community members.

### Appropriateness of content

In summary, 92% of all participants (both experts and community members combined) agreed that the malaria content and the SIT information in the song was useful and adequate. While they would have answered this from different perspectives, one group as content experts and the other as potential recipients of the SIT, they were in agreement that perceived information needs were met.

To further explore content effectiveness, community comments were analysed using open-ended questions to determine what participants remembered from the song and what they learned. Specifically, they were asked “*What information can you remember from the song*?” and “*Did you learn anything new from the song*? *If yes, what did you learn*?” These results were coded, grouped and quantified (see Table 3). This data supplemented information obtained from the six closed-ended questions that were posed to both experts and community members about the song content.

**Table 3:** Thematic areas and summary on what participants remembered and learned after listening to the song during the assessment of acceptability of a cultural song as a community engagement medium in KwaZulu-Natal Province, South Africa

Responses from the participants	Remembered n = 54 (%)	Learned n = 54 (%)
Female mosquitoes transmit malaria infection	18 (33)	8 (15)
Malaria can kill but is treatable	13 (24)	6 (11)
Male mosquitoes do not bite and do not transmit malaria	6 (11)	13 (24)
Sterilised male mosquito	3 (5)	5 (9)
Medication and indoor residual spraying	3 (5)	0
Signs and symptoms	2 (4)	5 (9)
Warning about malaria	6 (11)	1 (2)
Difference between male and female mosquito behaviour	3 (5)	10 (18)
SIT project	0	3 (5)
Seeking medical treatment if unwell	0	3 (5)

Three strong themes about malaria emerged when the community was probed on what they learned from the song. These were: malaria is fatal but treatable, male mosquitoes do not bite and the morphological difference between a male and female mosquito. Of these, the message that female mosquitoes transmit pathogens that cause infections was the most remembered theme (33%), followed by the fact that malaria can kill but is treatable (24%). Malaria signs and symptoms were least remembered, with fewer than 10% remembering this thematic area. One expert (MCP staff, Male 38) summed this by recommending: *“Expand on what is malaria and signs and symptoms.”*

The inclusion of information on mosquitoes being vectors for malaria was applauded by 97% of the experts. Repeated emphasis on the point that male mosquitoes do not bite in the song was hailed by 85% of the experts (Table 2), with 11% of community members spontaneously recalling this point (Table 3). This critical message was cited as a lesson learnt by 24% of the community members surveyed. About 18% participants from the community specified that they have learned about the difference in behaviour between male and female mosquitoes through the song, with 33% reporting that the song reminded them that only female mosquitoes transmit malaria (Table 3).

These views were also consistent with those expressed during CDS. In the CDS, participants expressed knowledge acquisition. One female participant summed:

“I gained a lot more information about malaria that I did not know before, including that only female mosquito transmit infection.” (Female 61 and Male 49)

The inclusion of SIT as an additional vector control method in the song was applauded by 87% of experts (Table 2). About 80% of the experts surveyed thought mentioning that SIT also targets outdoor mosquitoes was appropriate and that the song had enough information to help people understand the SIT technology. A higher proportion of community (96%) members also shared the same views. By contrast, when it came to retaining this information, fewer than 10% of the community remembered or reported learning about SIT in the open-ended questions.

In CDS, one participant noted:

“I have learned that male mosquito will be sterilized and released back into the wild, to help reduce the spread of malaria.” (Female 40)

### Overall appropriateness

Most experts (90%) and all community members (100%) agreed that the song was appropriate to engage the community about SIT.

In the CDS, participants noted:

“The song can really help communities and schools with malaria awareness and engagement. It is also useful to know that only female mosquito transmit infection.” (Female 36)

“This song should be played on national radio for everyone to understand and hear about the SIT project.” (Male 49)

The participants emphasized that this song should be played at schools, churches and the community, so that everyone can know and understand malaria and the SIT project, especially the young children. When asked if there was anything they could change about the song, all those who responded to this question (12%) agreed with this recommendation: *“Tone down the guitars so that back-up singers can be heard better”* (Females, ages 18–40).

## Discussion

Appropriateness is a multi-dimensional phenomenon. In this work appropriateness was taken in the context of whether a cultural song was acceptable and effective in conveying intended messages. Against this background, exploratory concurrent mixed methods were used to investigate the acceptability of using a cultural song in the “Maskandi” genre in three communities from Jozini, KwaZulu-Natal Province that fall within an area targeted for piloting the SIT technology. A local artist residing in the area was identified and worked with project members to generate or composition a song that will be used for community engagement purposes.

The findings of the study show that the community (both from the survey and community dialogue sessions) strongly agreed that the song was appropriate for age and culturally acceptable to engage and educate communities on malaria and the SIT technology. As the community was the intended audience of the music, their perspectives on cultural appropriateness should be weighted highly than those of SIT experts, who rated cultural appropriateness lowly compared to the community. The acceptability of using music to convey information has been noted in similar interventions, accounting in part for its widespread use in Africa [25, 28–40]. Overall participants’ response was positive; the song was well received and the audience reported that it was informative and entertaining. They were also pleased that one of their own (local artist) composed the song and the genre was the one that spoke to their tradition and reminded them of where they come from. This is in agreement with Ntombela^41^ who reported on the value Zulu people place on the “Maskandi” genre of music. This approach of using popular artists and working with the communities is commonly used in Africa, [29, 34–41] and increases the impact music has and ownership by the locals as observed from this study.

While the “Maskandi” genre was generally well received, the study found that this preference was not universal. This is not surprising and highlights the importance of using a tailored mix of communication approaches and channels to address audiences. Consequently, lessons from health communication scholars becomes relevant for community engagement activities. Specifically, communication channel choices should be guided by an understanding of the audience and their particular behavioural pathways [48]. Frameworks exist, such as the Audience—Channel—Message—Evaluation framework [49], that could support planning of community engagement efforts for maximum impact.

The community members further reported that the song was easy to remember, it allowed them to engage and that they benefitted from the repetition of verse. This finding is in agreement with Allan [27] that targeted songs or “jingles” can significantly increase audience ability to remember and understand the message.

The question of what was remembered and with what accuracy is critical to understanding how the audience engage with music. This is a key principle of audience reception research, which seeks to establish what kind of meanings audiences derive from communication and whether, based on their circumstances, these meanings have any effects (cognitive, social, political and/or emotional) [50]. One focus of this study was on what meaning audiences took from the song. The themes participants recalled, particularly about the links between mosquitoes and malaria and the differences between male and female mosquitoes, are promising, as the SIT project will be based on the mass release of sterile male mosquitoes. An understanding of these nuances may improve ultimate acceptability of the pilot project. A 2015 study by Manana et al. [43] found that 37% of study participants in KZN associated female mosquitoes as the carriers of infection. The current study suggests that exposure to the “Maskandi” song may reinforce and increase these knowledge levels.

Although knowledge that malaria can kill if not treated was spontaneously remembered by at least a quarter of the community participants and was the strongest theme, recall of malaria signs and symptoms was poor. In contrast, a knowledge, attitudes and practices (KAP) study by Manana and colleagues in the same communities reported 100% of the participants remembered that malaria can kill if untreated and 63% were able to recall at least three or four symptoms for malaria [43]. The previous study highlights a baseline of knowledge upon which the song could have reinforced existing knowledge in addition to introducing new ideas. The difference in results could be that this study post-coded open-ended questions whereas the KAP study used a quantitative survey, where the survey questions themselves may have prompted them to remember.

The song also emphasized that the SIT technology is not replacing the current vector control method (indoor residual spraying), but is an additional tool to compliment the current malaria control strategy [10]. Only 10% spontaneously reported to have remembered and learned about the SIT project. This finding is similar to the Manana et al*.* [43] study. More effort needs to be dedicated to improving awareness on the SIT technology through additional communication channels. This is important, as communities are still expected to comply with the national requirements regarding IRS as it is currently the main vector control strategy in South Africa [5, 6].

More practically, based on participant feedback, alterations can be made to the song to improve its effectiveness. The main recommendation focused on the audibility of the sound. Some members of the community and the expert group recommended that the song could be shortened and guitar sounds reduced so that the back-up voices could be heard well.

The study is not without limitations. As the survey sampling was not random across communities, the findings cannot be generalised. Furthermore, questions about what they remembered on the song were asked immediately after the song was played. This could have overestimated information retained. The qualitative component was done through community dialogues with large numbers of participants of mixed ages and genders, which may have resulted in some people feeling uncomfortable to share their views. Similarly, the format of data collection did not allow the same level of follow-up or probing as with other qualitative methods, such as focus group discussions or in-depth interviews. As such, the authors are unable to claim data saturation. Poor sound quality in the CDS recordings may have led to a loss of information during translation from isiZulu to English, or loss of meaning if note-takers missed details about the context of the dialogues. In retrospect, the use of focus group discussions, segmented by age and gender, could have mitigated these risks. Nevertheless, the use of mixed methods was a strength, as multiple sources of data enabled triangulation of results.

While the “Maskandi” experience is promising, whether increased knowledge about SIT will be sufficient to result in community acceptance of this technology remains to be seen. Fayoyin and Nieuwoudt [32] caution that enthusiasm for music as an enabler of change needs to be supported by empirical evidence, which is often lacking. As such, it is recommended that provisions are made for an impact evaluation of whether this and other efforts to engage with the Jozini communities results in greater community support for SIT.

## Conclusion

The “Maskandi” song developed was deemed educational, entertaining, culturally appropriate and informative in engaging community members on the SIT technology and its potential as a complementary vector control tool. It is concluded that cultural music is a potential tool that supports community engagement and if complimented with additional communication channels can reinforce and supplement the dissemination of key information. The recommendations made regarding the song will be considered and the current version updated and re-recorded as part of an ongoing effort to engage communities about SIT in KZN.

## Supplementary Information

**Additional file 1. **Malaria Song Lyrics

**Additional file 2. **Community Questionnaire

**Additional file 3. **Experts Questionnaire

## Data Availability

All data is available on reasonable request.
