# Supplementary material for: “Maskandi experience”: exploring the use of a cultural song for community engagement in preparation for a pilot Sterile Insect Technique release programme for malaria vector control in KwaZulu-Natal Province, South Africa 2019
Source: Malar J. 2021 Apr 28;20:204. doi: 10.1186/s12936-021-03736-9 (PMC8082840; doi:10.1186/s12936-021-03736-9)
Supplement: Supplementary file 1 — Additional file 1. Malaria Song Lyrics [file 12936_2021_3736_MOESM1_ESM.docx]

**Malaria Song Lyrics**

**Song:**

The most important thing to know about malaria disease

(Repeat x2)

What is malaria?

(Repeat x4)

The most important thing to know about malaria disease

Malaria is a fatal disease if it is not treated early

The good thing is: it is treatable and can be prevented

(Repeat x2)

How do people get malaria?

(Repeat x6)

People get malaria by being bitten by a female mosquito

A female mosquito is the one that spreads the malaria disease

How can you avoid getting malaria?

(Repeat x6)

Avoiding mosquito bites,

Taking medication if necessary and

Indoor residual spraying with effective medication

(Repeat x2)

All of this has put South Africa at a level to get rid of malaria

Therefore, additional strategies are needed to control malaria

Especially targeting mosquitoes that feed on the outside

(Repeat x2)

Sterile Insect Technique

Sterilization of male mosquitoes

(Repeat x2)

Sterile Insect Technique

Sterilization of male mosquitoes

(Repeat x2)

**Poet:**

Sterile Insect Technique

It is a method of birth control

that suppresses mosquito populations

and helps reduce malaria from spreading in our community

It is effective when applied together with other control methods

How does the Sterile Insect Technique work?

Mass rearing: mosquitoes are first mass reared

Sex sorting: mosquitoes are separated into males and females

only males are selected for release

Male mosquitoes do not bite and cannot transmit the disease

Male mosquitoes are sterilized using radiation that destroys the reproductive tract

After sterilization

mosquitoes have no reproductive power like the mosquitoes in the wild

Sterilized male mosquitoes are released in the wild and they mate with females,

who then produce sterile eggs

that bear no offspring

Key message: male mosquitoes do not bite

and will not infect you with malaria

Male mosquitoes are sterilized using radiation that destroys the reproductive tract

After sterilization

mosquitoes have no reproductive power like the mosquitoes in the wild

Sterilized male mosquitoes are released in the wild and they mate with females,

who then produce sterile eggs

that bear no offspring

This helps in reducing malaria from spreading in the area

South Africa let’s watch out for malaria

Down with malaria down….

Remember: male mosquitoes do not bite and will not infect you with malaria

**Song:**

Male mosquitoes are sterilised

And they are then released in the wild and they mate with females, who then produce sterile eggs that bear no offspring

(Repeat x2)

Sterile Insect Technique

Sterilization of male mosquitoes

(Repeat x2)

Sterile Insect Technique

Sterilization of male mosquitoes

(Repeat x2)

Malaria is a fatal disease if it is not treated early

The good thing is: it is treatable and can be prevented

(Repeat x2)

People get malaria by being bitten by an infective female mosquito

A female mosquito is the one that spreads the malaria disease
